# The ratio of serum eicosapentaenoic acid to arachidonic acid and risk of cancer death in a Japanese community: The Hisayama Study

**DOI:** 10.1016/j.je.2017.01.004

**Published:** 2017-06-29

**Authors:** Masaharu Nagata, Jun Hata, Yoichiro Hirakawa, Naoko Mukai, Daigo Yoshida, Tomoyuki Ohara, Hiro Kishimoto, Hiroyuki Kawano, Takanari Kitazono, Yutaka Kiyohara, Toshiharu Ninomiya

**Affiliations:** a Department of Epidemiology and Public Health, Graduate School of Medical Sciences, Kyushu University, Fukuoka, Japan; b Department of Medicine and Clinical Science, Graduate School of Medical Sciences, Kyushu University, Fukuoka, Japan; c Center for Cohort Studies, Graduate School of Medical Sciences, Kyushu University, Fukuoka, Japan; d Department of Neuropsychiatry, Graduate School of Medical Sciences, Kyushu University, Fukuoka, Japan; e Developmental Research, Mochida Pharmaceutical CO., Ltd., Gotenba, Japan; f Hisayama Research Institute For Lifestyle Diseases, Fukuoka, Japan

**Keywords:** Eicosapentaenoic acid, Arachidonic acid, Neoplasms, Mortality, Cohort studies

## Abstract

**Background:**

Whether the intake of eicosapentaenoic acid (EPA) or arachidonic acid (AA) affects the risk of cancer remains unclear, and the association between the serum EPA:AA ratio and cancer risk has not been fully evaluated in general populations.

**Methods:**

A total of 3098 community-dwelling subjects aged ≥40 years were followed up for 9.6 years (2002–2012). The levels of the serum EPA:AA ratio were categorized into quartiles (<0.29, 0.29–0.41, 0.42–0.60, and >0.60). The risk estimates were computed using a Cox proportional hazards model. The same analyses were conducted for the serum docosahexaenoic acid to arachidonic acid (DHA:AA) ratio and individual fatty acid concentrations.

**Results:**

During the follow-up period, 121 subjects died of cancer. Age- and sex-adjusted cancer mortality increased with lower serum EPA:AA ratio levels (*P* trend<0.05). In the multivariable-adjusted analysis, the subjects in the first quartile of the serum EPA:AA ratio had a 1.93-fold (95% confidence interval, 1.15–3.22) greater risk of cancer death than those in the fourth quartile. Lower serum EPA concentrations were marginally associated with higher cancer mortality (*P* trend<0.11), but the serum DHA or AA concentrations and the serum DHA:AA ratio were not (all *P* trend>0.37). With regard to site-specific cancers, lower serum EPA:AA ratio was associated with a higher risk of death from liver cancer. However, no such associations were detected for deaths from other cancers.

**Conclusions:**

These findings suggest that decreased level of the serum EPA:AA ratio is a significant risk factor for cancer death in the general Japanese population.

## Highlights

• Lower serum EPA:AA ratio was significantly associated with higher cancer mortality.
• The risk of death from liver cancer increased with lower serum EPA:AA ratio.
• No significant association was found between serum DHA:AA ratio and cancer death.

## Introduction

Omega-3 polyunsaturated fatty acids (PUFAs), such as eicosapentaenoic acid (EPA) and docosahexaenoic acid (DHA), must be obtained from foods because the amounts synthesized in the human body are very small.^1^ The main dietary sources of omega-3 PUFAs are marine fish and fish oil. Omega-3 PUFAs constitute an important component of human cell membranes and regulate inflammatory responses through the production of lipid mediators termed eicosanoids.^1^ Growing epidemiologic evidence suggests that the consumption of fish, fish oil, and omega-3 PUFAs protects against the development of cardiovascular diseases,^2^, ^3^ inflammatory diseases (e.g., rheumatoid arthritis and inflammatory bowel disease),^4^, ^5^ and mental illnesses (e.g., bipolar disorder and cognitive dysfunction).^6^, ^7^ Since some epidemiologic and small clinical studies have also reported that the intake of omega-3 PUFAs, including EPA, reduced the risk for the development or the recurrence of cancer,^8^, ^9^ the potential effect of omega-3 PUFAs on cancer risk has become a topic of scientific and public interest. However, this issue is still an area of controversy, because several population-based studies have shown conflicting results.^10^, ^11^, ^12^, ^13^, ^14^, ^15^, ^16^, ^17^, ^18^

Arachidonic acid (AA) is one of the sources of omega-6 PUFAs and a precursor of eicosanoids with parallel paths from EPA and DHA.^19^ AA is the substrate for the synthesis of a wide variety of eicosanoids, and is known to be proinflammatory, vasoconstrictive, and/or proaggregatory. On the other hand, it has also been shown that some AA-derived eicosanoids have anti-inflammatory and antiaggregatory effects.^20^, ^21^ Several basic science investigations reported that AA-derived eicosanoids have some carcinogenic effects, including cell proliferation,^22^, ^23^ apoptosis,^22^, ^23^ inflammation,^24^ and angiogenesis.^25^ However, epidemiologic evidence of the carcinogenic effects of AA remains unclear, due to the limited number of available studies and their methodological limitations; for instance, several observational studies failed to reveal a significant associations between AA exposure and cancer risk of some organs, such as the mammary glands and prostate.^26^ Meanwhile, eicosanoids derived from EPA and DHA have the opposite effects through their competition with AA.^19^ The ratios of serum EPA to AA (EPA:AA) or serum DHA to AA (DHA:AA) are thought to be good indicators of the balance between EPA or DHA and AA in the human body.^27^ We previously revealed that lower levels of the serum EPA:AA ratio, but not the serum DHA:AA ratio, were associated with a greater risk of cardiovascular disease.^28^ However, the associations between these indicators and the risk of cancer have not been fully evaluated in general populations.

The purpose of this study was to investigate whether lower levels of the EPA:AA ratio, as well as of the DHA:AA ratio, were associated with overall and site-specific cancer mortality in a 10-year follow-up study of a general Japanese population.

## Methods

### Study design and participants

The Hisayama Study is an ongoing, population-based epidemiologic study in the town of Hisayama, which is located in a suburb of the Fukuoka metropolitan area on Kyushu Island, Japan. The population of Hisayama is approximately 8400 and has been stable for 50 years, and the age and occupational distributions of the town population are almost identical to those of Japan as a whole.^29^ The rationale, study design, and methods of the Hisayama Study have been described elsewhere.^28^, ^30^ In 2002 and 2003, a screening examination for the present study was performed in the town. Briefly, a total of 3328 residents aged 40 years or older (participation rate, 77.6%) underwent the examination. After excluding 30 participants who did not consent to participate in the study, 6 without available data on serum fatty acid levels, and 194 with a history of cancer, the remaining 3098 subjects were enrolled in the study. This study was conducted with the approval of the Kyushu University Institutional Review Board for Clinical Research, and written informed consent was obtained from all subjects.

### Follow-up survey

The subjects were followed up prospectively for an average of 9.6 years, from the date of the screening examination to November 2012, using repeated health examinations and a daily monitoring system established by the study team and local physicians or members of the town's Health and Welfare Office. For any subject who did not undergo an annual examination or who moved out of town, the health status was checked via mail or telephone every year. Information about death was gathered through this follow-up system. When a subject died, we collected all medical information related to his/her illness and death, including hospital charts, physicians' records, and death certificates. Moreover, an autopsy was performed in the Department of Pathology at Kyushu University, if consent for an autopsy was obtained.^31^, ^32^ All subjects were followed up completely until the end of the survey. During the follow-up period, 421 subjects died, of whom 263 (62.5%) underwent autopsy.

### Measurement of risk factors

At baseline, blood samples were collected from an antecubital vein, and a portion of the serum was stored at −80 °C until use for the measurement of serum fatty acids concentrations and serum high-sensitivity C-reactive protein (HS-CRP) levels in 2010.^28^ Serum fatty acids levels were assayed using gas chromatography^33^ (SRL, Tokyo, Japan). Briefly, total lipids in plasma were extracted according to Folch's procedure,^34^ followed by hydrolysis to free fatty acids. Free fatty acids were esterified with potassium methoxide/methanol and boron trifluoride-methanol. The methylated fatty acids were analysed using a GC-17A gas chromatograph (Shimadzu Corporation, Kyoto, Japan) with an Omegawax-250 capillary column (Supelco, Sigma–Aldrich Japan, Tokyo, Japan). Tricosanoic acid (C23:0) was used as an internal standard substance. The reproducibility of this method is expressed using the intra-assay percent coefficient of variation (%CV) from four samples. The %CV of the serum EPA, DHA, and AA levels was reported to be 4.4%, 2.3%, and 3.8%, respectively.^33^ Serum HS-CRP levels were measured using a modification of the Behring Latex-Enhanced CRP assay on a BN-100 nephelometer (Behring Diagnostics, Westwood, MA, USA). At the screening examination, plasma glucose levels were measured using the glucose oxidase method. Diabetes mellitus was defined as a fasting plasma glucose level of ≥7.0 mmol/L (126 mg/dL), 2-h post-loaded or casual glucose level of ≥11.1 mmol/L (200 mg/dL), or current use of insulin or oral glucose-lowering agents. Serum total cholesterol and high-density lipoprotein (HDL) cholesterol concentrations were determined enzymatically. Serum non-HDL-cholesterol levels were calculated by subtracting HDL-cholesterol from total cholesterol values.

A self-administered questionnaire concerning history of cancer, current use of anti-hypertensive agents, insulin, oral glucose-lowering agents, lipid-modifying agents, and agents containing EPA, in addition to smoking habits, drinking habits, and regular exercise was checked by trained interviewers. Smoking habits and drinking habits were classified as either current use or not. Those subjects engaging in sports or other forms of exertion three or more times per week during their leisure time made up a regular exercise group. Hypertension was defined as blood pressure ≥140/90 mm Hg or current use of anti-hypertensive agents. Height and body weight were measured in light clothes without shoes, and body mass index (kg/m^2^) was calculated.

### Definition of cancer death

All of the available medical data, including autopsy findings, were reviewed for the deceased cases, and the underlying causes of death were determined and classified according to the International Classification of Diseases, Tenth Revision (ICD-10). In addition, the category of cancer death (ICD-10 code C00-99) was sub-classified into six site-specific cancers of lung (C31.0–34.9 and C06.0), stomach (C13.9, C15.0–15.9, and C16.0–16.9), colorectum (C17.0–17.9 and C18.0–20.0), pancreas (C25.0–25.9), or other sites. All cancer deaths were adjudicated on the basis of physical examination; a review of all clinical data, including medical records; and autopsy findings by a panel of the study members, who remained blind to the information on each subject's serum fatty acids levels. During the follow-up period, 121 subjects (73 men and 48 women) died of cancer, and deaths due to other causes were censored at the date of death.

### Statistical analysis

The serum EPA:AA ratio levels were divided into quartiles (<0.29, 0.29–0.41, 0.42–0.60, and >0.60). The linear trends in the mean values and frequencies of risk factors for cancer death across the serum EPA:AA levels were tested using linear regression analysis and logistic regression analysis, respectively, with evenly spaced numeric codes (i.e., 1, 2, 3, and 4) for the serum EPA:AA levels. The age- and sex-adjusted mortality from cancer was calculated by the person-years method and adjusted for the age and sex distribution of the overall study population using the direct method.^35^ A Cox proportional hazards model was used to estimate the adjusted hazard ratio (HR) with 95% confidence intervals (CIs) of the outcomes according to serum EPA:AA ratio levels. In the multivariable-adjusted model, the adjustment was made for clinically or biologically plausible risk factors for the outcomes — namely, age, sex, hypertension, diabetes, serum HDL cholesterol, serum non-HDL cholesterol, use of lipid-modifying agents, body mass index, serum HS-CRP, smoking habits, drinking habits, and regular exercise. The analyses of the serum DHA:AA ratio and serum EPA, DHA, and AA concentrations were conducted in the same manner, separately. Non-linear association between the serum EPA:AA ratio and the risk of cancer mortality was tested using a relevant Cox model including spline terms with three knots of quartile values (serum EPA:AA ratio: 0.29, 0.41, and 0.60). To check the possibility of the existence of subclinical cancer at baseline, we performed a sensitivity analysis of the association between the serum EPA:AA ratio and the risk of cancer death after excluding the first 2 years of follow-up. The SAS software package, version 9.3 (SAS Institute, Cary, NC, USA), was used to perform all statistical analyses. Two-sided values of *P* < 0.05 were considered statistically significant in all analyses.

## Results

The baseline characteristics of the study subjects according to the quartiles of the serum EPA:AA ratio are summarized in Table 1. Older men showed higher serum EPA:AA ratios. Compared with subjects with lower serum EPA:AA ratios, the median values of serum EPA and DHA were significantly higher in those with higher serum EPA:AA ratios, but no such associations were observed for serum AA. In addition, subjects with higher serum EPA:AA ratios were more likely to have hypertension, diabetes, higher serum non-HDL cholesterol, a habit of using EPA-containing agents, higher body mass index, a drinking habit, and regular exercise, but no such associations were observed for serum HDL cholesterol, HS-CRP, or smoking habit. We observed similar associations between the serum DHA:AA ratio levels and other risk factors at baseline (eTable 1).

**Table 1:** Baseline characteristics of the study participants by quartiles of serum EPA:AA ratio.

Variables	Serum EPA:AA ratio				*P* for trend
	<0.29 (*n* = 775)	0.29–0.41 (*n* = 774)	0.42–0.60 (*n* = 775)	>0.60 (*n* = 774)	
Age, years	58.8 (14.3)	60.7 (12.7)	62.2 (11.8)	63.7 (10.7)	<0.001
Men, %	36.5	38.2	39.2	55.7	<0.001
Serum EPA, μg/mL	32.4 (24.5–39.8)	53.1 (43.9–62.2)	72.9 (60.6–86.5)	112.0 (91.1–139.6)	<0.001
Serum DHA, μg/mL	101.3 (80.5–123.6)	129.1 (107.2–154.2)	145.8 (123.5–176.3)	175.6 (148.0–215.8)	<0.001
Serum AA, μg/mL	150.0 (125.8–178.7)	151.2 (126.7–174.2)	148.0 (125.4–172.5)	144.0 (123.8–166.7)	<0.001
Systolic blood pressure, mm Hg	128 (21)	131 (21)	133 (20)	136 (22)	<0.001
Diastolic blood pressure, mm Hg	77 (12)	78 (12)	79 (12)	80 (12)	<0.001
Use of anti-hypertensive agents, %	16.7	24.9	24.1	30.0	<0.001
Hypertension, %	34.7	43.0	45.3	53.2	<0.001
Diabetes, %	11.6	14.9	15.6	23.8	<0.001
Serum total cholesterol, mmol/L	5.18 (0.97)	5.27 (0.86)	5.40 (0.87)	5.28 (0.93)	0.003
Serum HDL cholesterol, mmol/L	1.60 (0.41)	1.61 (0.43)	1.65 (0.42)	1.61 (0.42)	0.22
Serum non-HDL cholesterol, mmol/L	3.59 (0.98)	3.66 (0.87)	3.76 (0.90)	3.67 (0.94)	0.02
Use of lipid-modifying agents, %	7.1	10.3	10.6	10.7	0.02
Use of agents containing EPA, %	0.1	0.0	0.0	2.1	<0.001
Body mass index, kg/m^2^	22.5 (3.6)	22.9 (3.3)	23.4 (3.4)	23.6 (3.2)	<0.001
Serum HS-CRP, mg/L	0.46 (0.20–1.03)	0.49 (0.24–1.02)	0.46 (0.23–1.03)	0.48 (0.26–1.11)	0.05
Smoking habits, %	23.1	20.9	20.0	24.3	0.69
Drinking habits, %	36.4	39.0	44.4	54.3	<0.001
Regular exercise, %	8.8	10.9	9.2	13.1	0.02

AA, arachidonic acid; DHA, docosahexaenoic acid; EPA, eicosapentaenoic acid; HDL, high-density lipoprotein; HS-CRP, high-sensitivity C-reactive protein.

Values are means (standard deviations), medians (interquartile ranges) or frequencies.

**Fig. 1:**
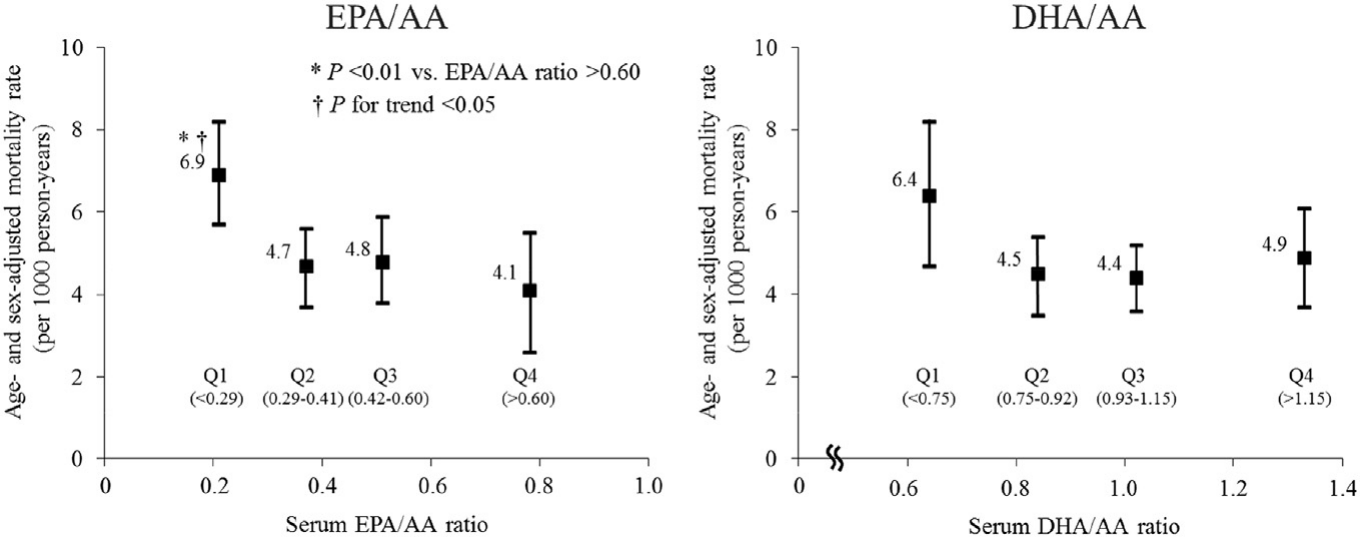
Age- and sex-adjusted mortality rate from cancer according to the quartiles of the serum EPA:AA and the DHA:AA ratios. The vertical bars represent 95% confidence intervals of the mortality rate. AA, arachidonic acid; DHA, docosahexaenoic acid; EPA, eicosapentaenoic acid.

Fig. 1 shows the age- and sex-adjusted mortality from cancer according to the quartiles of the serum EPA:AA ratio or the serum DHA:AA ratio. The age- and sex-adjusted cancer mortality rate increased with lower serum EPA:AA ratio levels, and a significant difference was observed between the first and fourth quartiles of the serum EPA:AA ratio (6.9 vs. 4.1 per 1000 person-years, *P* < 0.01), but no such association was observed for the serum DHA:AA ratio. These associations were substantially unchanged after adjustment for the aforementioned confounding factors. The risk of death from cancer was significantly higher in subjects with a serum EPA:AA ratio in the first quartile than in those with serum EPA:AA in the fourth quartile (multivariable-adjusted HR 1.93; 95% CI, 1.15–3.22; Table 2). In addition, we tested the non-linear association between the serum EPA:AA ratio and the risk of cancer mortality using spline models, but significant changes in the slope were not observed between the first and the second, the second and the third, or the third and the fourth quartiles of the serum EPA:AA ratio (all *P* > 0.10).

**Table 2:** Hazard ratios for death from cancer according to quartiles of serum EPA:AA and DHA:AA ratios.

	Number of events	Number of subjects	Age- and sex-adjusted			Multivariable-adjusted^a^		
			HR (95% CI)	*P* value	*P* for trend	HR (95% CI)	*P* value	*P* for trend
Serum EPA:AA ratio								
Q4 (>0.60)	28	774	1.00 (reference)		0.01	1.00 (reference)		0.02
Q3 (0.42–0.60)	29	775	1.27 (0.76–2.15)	0.36		1.34 (0.79–2.27)	0.27	
Q2 (0.29–0.41)	29	774	1.35 (0.80–2.27)	0.26		1.34 (0.79–2.28)	0.27	
Q1 (<0.29)	35	775	1.94 (1.18–3.20)	0.009		1.93 (1.15–3.22)	0.01	
Serum DHA:AA ratio								
Q4 (>1.15)	36	774	1.00 (reference)		0.29	1.00 (reference)		0.37
Q3 (0.93–1.15)	31	775	1.01 (0.62–1.63)	0.97		1.03 (0.63–1.67)	0.91	
Q2 (0.75–0.92)	27	775	1.00 (0.61–1.65)	0.99		1.02 (0.61–1.69)	0.95	
Q1 (<0.75)	27	774	1.38 (0.84–2.27)	0.21		1.32 (0.79–2.21)	0.30	

AA, arachidonic acid; CI, confidence interval; DHA, docosahexaenoic acid; EPA, eicosapentaenoic acid; HR, hazard ratio.

Use of lipid-modifying agents, body mass index, serum high-sensitivity C-reactive protein, smoking habits, drinking habits, and regular exercise.

a Adjusted for age, sex, hypertension, diabetes, serum high-density lipoprotein cholesterol, serum non-high-density lipoprotein cholesterol.

To exclude the influence of baseline subclinical cancers on these findings, we performed sensitivity analysis after excluding cases of cancer death developed in the first 2 years of follow-up. The significant association between the serum EPA:AA ratio and the risk of cancer death was still observed after adjusting for the aforementioned confounding factors (first vs. fourth quartile: HR 1.84; 95% CI, 1.07–3.18), but no such association was found for the serum DHA:AA ratio (HR 1.26; 95% CI, 0.71–2.23). We also analyzed our data using serum concentrations of EPA, DHA, and AA separately (eTable 2). Subjects with lower serum EPA concentrations tended toward having higher risk of cancer death, but this association did not reach the statistically significant level. In addition, there were no clear associations between serum AA levels and the risk of cancer death.

Finally, we examined the association between the serum EPA:AA ratio and the risk of site-specific cancer death (Table 3). The age- and sex-adjusted HR for death from liver cancer increased significantly in subjects with a serum EPA:AA ratio in the first quartile compared with subjects with a serum EPA:AA in the second–fourth quartiles (HR 4.59; 95% CI, 1.40–15.10), but no clear associations were seen between the serum EPA:AA ratio and death from cancers of the lung, stomach, colorectum, pancreas, or other sites.

**Table 3:** Risk of site-specific cancer death in subjects with low levels of serum EPA:AA ratio.

Site-specific cancer	Number of events/subjects	HR (95% CI) of serum EPA:AA ratio of <0.29^a^ (vs. serum EPA:AA ratio of ≥0.29)	*P* value
Lung cancer	25/3098	1.94 (0.84–4.52)	0.12
Stomach cancer	18/3098	1.16 (0.38–3.53)	0.80
Colorectal cancer	14/3098	1.16 (0.38–3.53)	0.46
Liver cancer	11/3098	4.59 (1.40–15.10)	0.01
Pancreatic cancer	8/3098	0.55 (0.07–4.45)	0.57
Other cancer	45/3098	1.46 (0.75–2.83)	0.27

AA, arachidonic acid; CI, confidence interval; EPA, eicosapentaenoic acid; HR, hazard ratio.

a The risk estimates were adjusted for age and sex.

## Discussion

Our prospective study of a general Japanese population suggests that lower levels of the serum EPA:AA ratio are associated with an increased risk of cancer death. This association remained robust even after adjustment for other confounding risk factors. In particular, the risk of death from liver cancer significantly increased with lower levels of the serum EPA:AA ratio. By contrast, we found no clear evidence of an association between the serum DHA:AA ratio and cancer death. To the best of our knowledge, this is the first report to show that decreased serum EPA:AA levels are an independent risk factor for cancer death. These findings suggest that the regular intake of EPA-rich foods may be effective for reducing the risk of cancer in the general Japanese population.

Although experimental models have consistently shown a modulation of carcinogenesis via omega-3 PUFAs,^36^, ^37^ epidemiologic studies have reported conflicting results on the association between omega-3 PUFAs intake and cancer risk. In several longitudinal observational studies, inverse associations were found between dietary intake of fish or omega-3 PUFAs and risk of some site-specific cancers, such as colon, breast, lung, and liver cancers,^10^, ^11^, ^12^, ^13^, ^14^ but other studies did not show these significant associations.^15^, ^16^, ^17^, ^18^ Recent systematic reviews have provided limited evidence of a possible role of dietary omega-3 PUFAs in preventing colon cancer, but failed to reveal any conclusive evidence, because the findings across studies were heterogeneous.^38^ The heterogeneity of these findings may have derived from the inherent difficulties of epidemiology (e.g., measurement error, relevance of biomarkers, and genetic background) and the differences in the dietary patterns and intake amounts of foods and nutrients among races.^38^ In addition, the heterogeneous findings may also be explained through genetic polymorphism and confounding with the nutrients from other foods (e.g., meat products or processed foods).^38^ Intriguingly, the results from Asian studies have shown favorable and homogenous findings of the effects of omega-3 PUFAs on cancer risk,^12^, ^13^, ^14^ in accordance with our present results. These findings may be attributable to an underlying favorable dietary pattern (e.g., higher intake of fish and leafy vegetables and lower intake of meat and animal fat) in Asian countries.^39^, ^40^ Further investigations will be necessary to elucidate this issue.

Several mechanisms may account for the potential preventive effects of elevated serum EPA:AA ratio levels against carcinogenesis and cancer proliferation. Recent data have expanded the concept that inflammation is a critical component of cancer incidence and progression.^41^ EPA plays an important role in suppression of the inflammatory responses by competing with AA.^42^, ^43^ EPA gives rise to a different family of eicosanoid mediators, including the 3-series prostaglandin and leucotriene B_5_, which are considered to be less inflammatory than AA-derived eicosanoids.^44^, ^45^ In addition, AA-derived eicosanoids themselves promote cancer cell growth and progression via several biological processes, including carcinogenic activity,^46^ inhibition of apoptosis,^47^ promotion of angiogenesis,^25^ and cancer cell adhesion,^48^ whereas EPA exerts other anticarcinogenic effects through a reduction in the production of free radicals and reactive oxygen species,^49^ an increment of insulin sensitivity,^50^ and an alteration of estrogen metabolism.^19^ These findings support the hypothesis that the balance between EPA and AA is important for regulating the production of mediators and subsequent carcinogenesis and cancer proliferation. Moreover, EPA may be of importance for cancer development, because EPA, but not AA, tended to be associated with cancer risk.

With regard to site-specific cancers, we found that lower serum EPA:AA ratios were significantly related with the risk of death from liver cancer. Some epidemiologic studies have shown an inverse association between the consumption of fish or omega-3 PUFAs and the risk of liver cancer.^13^, ^14^ A recent prospective study including 90,000 Japanese subjects, which estimated the consumption of fish or omega-3 PUFAs from self-reports, has demonstrated that the incidence of liver cancer decreased with higher intake of fish or omega-3 PUFAs.^14^ Since chronic inflammation of the liver, which is triggered by hepatitis virus infection, alcoholic abuse, or autoimmunity,^51^ is a well-known cause of liver cirrhosis and subsequent liver cancer, EPA may have a beneficial effect against liver cancer in individuals under chronic inflammatory conditions. The present study, however, failed to reveal any significant association between the serum EPA:AA ratio and the risk of death from other organ cancers. The exact reasons for the difference in the influence of EPA among site-specific cancers are not clear, but they may be related to the different etiology or malignancy of the cancer among organs.

In contrast to the serum EPA:AA ratio, we found no significant inverse association between the serum DHA:AA ratio and the risk of cancer death in the present study. The biologic mechanisms underlying the difference between the serum EPA:AA ratio and the DHA:AA ratio are unclear. However, EPA may have more preventive effects against cancer than DHA through the suppression of inflammatory responses and promotion of apoptosis in cancer cells.^52^, ^53^ In support of this notion, the present study showed that the risk of cancer death tended to increase with lower serum EPA concentrations, but there was no clear association between the serum DHA or AA concentrations and cancer mortality (eTable 2). These results suggest that the observed association between the serum EPA:AA ratio and the risk of cancer death is mainly driven by serum EPA concentrations. Further epidemiologic and experimental studies addressing the differences in the effects among EPA, DHA, and AA will be required.

The strengths of our study include its longitudinal population-based design, low selection bias, perfect follow-up of subjects, and accurate diagnosis of the cause of death on the basis of medical records and autopsy findings. Some limitations of this study should be noted. First, the evaluation of serum PUFA levels was based on a single measurement at baseline, as is the case in most epidemiologic studies. Furthermore, there is a possibility that serum PUFA levels reflect only recent dietary consumption. These limitations could have caused a misclassification of study subjects into various categories and thereby underestimated the true associations. Second, the existence of subclinical cancer at baseline was undeniable, because no screening survey for cancer was performed at screening. However, the sensitivity analysis, in which we excluded cancer deaths occurring in the first 2 years of follow-up, did not make any material difference in the findings, suggesting that the influence of this limitation would have been small. Fourth, we were unable to obtain information about medical treatment during the follow-up period. The lack of such information may have reduced the accuracy of our findings to some extent. Finally, the significance of our findings may be limited because of the small number of cancer deaths among those surveyed.

In conclusion, the present analysis showed that lower levels of the serum EPA:AA ratio, but not the serum DHA:AA ratio, were significantly associated with a greater risk of cancer death. These associations suggest that lower serum EPA:AA ratios are a possible risk factor for cancer death. Notably, the serum EPA:AA ratio may relate to the risk of death from liver cancer. These findings imply that the regular intake of EPA-rich foods may be effective for reducing the risk of cancer. Further large-scale prospective cohort studies will be needed to confirm the influence of the serum EPA:AA ratio on the risk of cancer death.

## Conflicts of interest

I am currently conducting research sponsored by Mochida Pharmaceutical Company and am also a member of the speakers for Takeda Pharmaceutical Company and Mochida Pharmaceutical Company.

## Funding support

This study was supported in part by Grants-in-Aid for Scientific Research (A) (25253048) and (C) (25460758, 26350895, 26460748, 15K09267, 15K08738, and 15K09835) from the Ministry of Education, Culture, Sports, Science and Technology of Japan; by Health and Labour Sciences Research Grants of the Ministry of Health, Labour and Welfare of Japan (H25-Junkankitou [Seishuu]-Sitei-022, H26-Junkankitou [Seisaku]-Ippan-001, and H27-Shokuhin-[Sitei]-017); and by the Japan Agency for Medical Research and Development (AMED) (15dk0207003h0003, 15dk0207018h0001, 15ek0210001h0003, 15ek0210004h0102, and 15gm0610007h0203 (CREST)). In addition, this study was sponsored by Mochida pharmaceutical Co., Ltd. (Tokyo, Japan). The sponsor of the study had no role in the study design, conduct of the study, data collection, or preparation of the report.
