# Supplementary material for: The ratio of serum eicosapentaenoic acid to arachidonic acid and risk of cancer death in a Japanese community: The Hisayama Study
Source: J Epidemiol. 2017 Jun 29;27(12):578–83. doi: 10.1016/j.je.2017.01.004 (PMC5623032; doi:10.1016/j.je.2017.01.004)
Supplement: Supplementary file 1 [file mmc1.pdf]

eTable 1. Baseline characteristics of the study participants by quartile of serum DHA:AA ratio

Variables	Serum DHA:AA ratio				*P* for trend
	<0.75 (n=775)	0.75-0.92 (n=774)	0.93-1.15 (n=775)	>1.15 (n=774)	
Age, years	57.1 (13.4)	60.5 (13.0)	62.9 (11.5)	65.0 (10.7)	<0.001
Men, %	38.2	38.8	39.5	53.1	<0.001
Serum EPA, µg/mL	37.3 (27.2-52.5)	55.3 (41.2-75.6)	70.9 (52.7-95.1)	94.4 (68.5-125.4)	<0.001
Serum DHA, µg/mL	98.0 (80.0-114.9)	126.9 (106.0-149.4)	146.3 (127.9-171.0)	189.1 (160.4-228.9)	<0.001
Serum AA, µg/mL	156.1 (132.7-183.6)	152.7 (128.3-175.8)	144.4 (125.4-165.1)	139.6 (119.5-163.5)	<0.001
Systolic blood pressure, mmHg	127 (21)	131 (20)	132 (21)	138 (21)	<0.001
Diastolic blood pressure, mmHg	76 (12)	78 (12)	78 (11)	81 (12)	<0.001
Use of anti-hypertensive agents, %	16.3	22.7	25.0	31.7	<0.001
Hypertension, %	32.3	42.5	45.3	56.2	<0.001
Diabetes, %	10.6	13.9	16.0	25.3	<0.001
Serum total cholesterol, mmol/L	5.17 (0.96)	5.30 (0.89)	5.37 (0.88)	5.30 (0.91)	0.003
Serum HDL cholesterol, mmol/L	1.68 (0.43)	1.65 (0.42)	1.64 (0.42)	1.49 (0.39)	<0.001
Serum non-HDL cholesterol, mmol/L	3.50 (0.96)	3.64 (0.90)	3.73 (0.88)	3.80 (0.92)	<0.001
Use of lipid-modifying agents, %	8.5	9.8	10.1	10.3	0.23
Use of agents containing EPA, %	0.5	0.9	0.4	0.4	0.45
Body mass index, kg/m^2	22.5 (3.6)	23.1 (3.7)	23.1 (3.1)	23.7 (3.3)	<0.001
Serum HS-CRP, mg/L	0.44 (0.19-0.95)	0.45 (0.21-0.96)	0.48 (0.23-1.03)	0.57 (0.30-1.29)	0.30
Smoking habits, %	24.4	19.7	19.2	24.9	0.88
Alcohol intake, %	41.1	41.2	41.6	50.3	<0.001
Regular exercise, %	8.0	10.2	9.9	13.7	<0.001

AA, arachidonic acid; DHA, docosahexaenoic acid; EPA, eicosapentaenoic acid; HDL, high-density lipoprotein; HS-CRP, high-sensitivity C-reactive protein.

Values are means (standard deviations), medians (interquartile ranges), or frequencies.

eTable 2. Hazard ratios for death from cancer according to serum EPA, DHA, and AA concentrations

	Number of events	Number of subjects	Age- and sex-adjusted			Multivariable-adjusted[a]			Multivariable-adjusted[b]		
			HR (95% CI)	*P* value	*P* for trend	HR (95% CI)	*P* value	*P* for trend	HR (95% CI)	*P* value	*P* for trend
Serum EPA concentration, μmol/L											
Q4 (>89.4)	26	774	1.00 (reference)		0.06	1.00 (reference)		0.10	1.00 (reference)		0.11
Q3 (61.6-89.4)	32	776	1.41 (0.84-2.37)	0.20		1.45 (0.86-2.46)	0.17		1.48 (0.86-2.55)	0.16	
Q2 (41.9-61.5)	31	775	1.40 (0.83-2.37)	0.21		1.40 (0.81-2.41)	0.22		1.43 (0.81-2.55)	0.22	
Q1 (<41.9)	32	774	1.70 (1.01-2.86)	0.04		1.68 (0.95-3.00)	0.08		1.77 (0.94-3.35)	0.08	
Serum DHA concentration, μmol/L											
Q4 (>171.9)	29	774	1.00 (reference)		0.60	1.00 (reference)		0.91	1.00 (reference)		0.99
Q3 (137.9-171.9)	28	775	0.85 (0.52-1.40)	0.53		0.86 (0.52-1.43)	0.56		0.85 (0.50-1.44)	0.54	
Q2 (107.6-137.8)	29	775	0.93 (0.57-1.54)	0.78		0.89 (0.52-1.52)	0.67		0.88 (0.50-1.55)	0.65	
Q1 (<107.6)	35	774	1.14 (0.70-1.87)	0.60		1.03 (0.59-1.81)	0.92		0.99 (0.53-1.85)	0.98	
Serum AA concentration, μmol/L											
Q4 (>172.6)	36	774	1.00 (reference)		0.88	1.00 (reference)		0.68	1.00 (reference)		0.75
Q3 (148.3-172.6)	30	775	0.78 (0.46-1.32)	0.36		0.75 (0.43-1.30)	0.30		0.75 (0.43-1.32)	0.32	
Q2 (125.4-148.2)	26	775	0.95 (0.57-1.58)	0.83		0.86 (0.50-1.49)	0.59		0.88 (0.50-1.55)	0.66	
Q1 (<125.4)	29	774	0.97 (0.59-1.59)	0.90		0.82 (0.45-1.48)	0.50		0.83 (0.45-1.54)	0.55	

AA, arachidonic acid; CI, confidence interval; DHA, docosahexaenoic acid; EPA, eicosapentaenoic acid; HDL, high-density lipoprotein; HS-CRP, high-sensitivity C-reactive protein; HR, hazard ratio.

[a] Adjusted for age, sex, hypertension, diabetes, serum HDL cholesterol, serum non-HDL cholesterol, use of lipid-modifying agents, body mass index, HS-CRP, current smoking, current drinking, and regular exercise.

[b] Adjusted for age, sex, hypertension, diabetes, serum HDL cholesterol, serum non-HDL cholesterol, use of lipid-modifying agents, body mass index, HS-CRP, current smoking, current drinking, regular exercise, saturated even chain fatty acids, monounsaturated fatty acid, omega-3 polyunsaturated fatty acids (excluding in the analysis of serum EPA and DHA concentrations), and omega-6 polyunsaturated fatty acids (excluding in the analysis of serum AA concentration).
